# A novel stiffness-distribution-controlled hybrid locking compression plate for osteoporotic midshaft clavicle fractures: a finite element study

**DOI:** 10.1186/s12891-026-10355-5

**Published:** 2026-08-14

**Authors:** Hui-Gyeong Gong, Jae-Soo Kim, Hyun-Ju Lee, Ki-Sik Tae

**Affiliations:** 1 Department of Biomedical Engineering, Konyang University, Daejeon, Republic of Korea; 2 Department of Orthopedic Surgery, Chung-Ang University Gwangmyeong Hospital, Gwangmyeong, Gyeonggi-do, Republic of Korea; 3 Department of Physical Therapy, Konyang University, Daejeon, Republic of Korea

**Keywords:** Osteoporotic Midshaft clavicle fractures, Stiffness-distribution–controlled hybrid locking compression plate (Hybrid LCP), Finite element analysis, Cancellous bone strain, Stiffness modulation

## Abstract

**Background:**

Fixation of osteoporotic midshaft clavicle fractures in elderly patients requires a careful balance between mechanical integrity and anatomical compatibility. Conventional single-diameter locking plate systems present an inherent trade-off, as larger plates provide robust fixation but often cause soft tissue irritation, whereas low-profile plates often lack the mechanical integrity in osteoporotic bone. This study aimed to biomechanically evaluate a stiffness-distribution–controlled hybrid locking compression plate (Hybrid LCP) designed to mitigate this trade-off using a patient-derived finite element analysis.

**Methods:**

A three-dimensional finite element model of a clavicle was reconstructed from CT data of a 73-year-old female, incorporating severe osteoporotic cortical thinning (1.0 mm). A Hybrid LCP combining a 3.5-mm central segment with 2.7-mm lateral segments was compared with conventional 3.5-mm and 2.7-mm plates under cantilever bending, axial compression, and axial torsion. Plate stress and peak cortical and cancellous bone stress-strain responses were evaluated to assess structural integrity and the risk of localized bone failure.

**Results:**

Cantilever bending produced the highest stress levels among all loading scenarios. Under this condition, the maximum von Mises stress in the Hybrid LCP (453.4 MPa) was lower than those of the 2.7-mm systems and the conventional 3.5-mm plate (629.5 MPa), indicating a more favorable plate stress distribution. Also, the Hybrid LCP exhibited 50.4% lower peak cancellous bone strain than the conventional curved 2.7-mm plate (6,300 µε vs. 12,700 µε).

**Conclusion:**

Under the standardized computational conditions evaluated in this study, the stiffness-distribution–controlled Hybrid LCP reduced stress concentrations associated with low-profile plates and decreased peak cancellous bone strain compared with the curved 2.7-mm plate. These findings suggest that targeted stiffness modulation may provide a promising biomechanical design approach for osteoporotic midshaft clavicle fixation. Further experimental and clinical validation is required before its clinical applicability can be established.

## Introduction

Midshaft clavicle fractures represent the most common injury of the shoulder girdle, accounting for approximately 80% of all clavicular fractures [1, 2]. Although nonoperative treatment was historically favored, accumulating clinical evidence has demonstrated unacceptably high rates of malunion and nonunion following conservative management, particularly in displaced fractures [3, 4]. As a result, surgical fixation has become increasingly recommended to enable early functional recovery and return to daily activities. Among available fixation methods, locking compression plates (LCPs) offer superior resistance to bending and torsional loads and are currently regarded as the standard of care for midshaft clavicle fractures [5, 6].

Concurrently, the epidemiology of clavicle fractures has shifted markedly, with a rapidly increasing incidence observed in elderly populations [7]. Fixation in elderly patients poses distinct biomechanical challenges due to age-related deterioration in bone morphology and quality. Elderly clavicles exhibit pronounced anatomical variability, complex three-dimensional curvature, and progressive loss of bone mineral density that continues beyond the age of 65 [8–10]. Among these factors, cortical bone thinning is particularly critical. Anatomical studies have demonstrated that cortical thickness decreases significantly in elderly individuals, potentially reaching as low as 1.0 mm in severe osteoporotic cases [11, 12]. This geometric vulnerability is not merely an anatomical variation but serves as a dominant failure mechanism, directly compromising screw purchase and increasing the risk of pull-out in osteoporotic fixation [13–15].

Despite these challenges, currently available commercial clavicle plating systems are not specifically optimized for elderly bone conditions. While conventional 3.5-mm systems provide robust fixation, their bulkiness poses risks of soft tissue irritation and stress shielding in the thin clavicles of elderly patients [16–19]. Conversely, low-profile 2.7-mm systems offer better anatomical fit but often lack the mechanical integrity required for unstable osteoporotic fractures [20, 21], creating a fundamental trade-off between construct rigidity and anatomical compatibility, which highlights the limitations of single-diameter plate systems in elderly clavicle fracture fixation [22].

To overcome these limitations, implant designs tailored to the biomechanical constraints of elderly bone are required. Finite element analysis (FEA) has been extensively applied to investigate plate stiffness, von Mises stress distribution, and construct mechanical behavior in clavicle fixation [23–29]. However, most prior studies have focused primarily on global stiffness metrics or von Mises stress distributions, which provide limited insight into clinically relevant failure mechanisms such as screw loosening and pull-out. These failure modes are more directly governed by axial tensile and compressive stresses acting along the screw insertion axis rather than von Mises stress measures alone [30].

Accordingly, the present study introduces a stiffness-distribution–controlled Hybrid LCP that strategically combines a rigid 3.5-mm central segment with low-profile 2.7-mm lateral segments to address the competing mechanical demands of elderly clavicle fixation. A patient-derived finite element model incorporating a cortical thickness of 1.0 mm was constructed to simulate severe osteoporotic thinning. To place this condition into clinical perspective, Andermahr et al. reported a mean midshaft cortical thickness of 2.05 ± 0.29 mm in normal clavicles [12]. Therefore, the cortical thickness of 1.0 mm evaluated in this study represents an approximate 50% reduction compared with the reported normal value and was selected to represent a clinically relevant worst-case osteoporotic condition. Unlike conventional analyses that focus primarily on overall construct stiffness or a single peak von Mises stress value, this study employed a screw-specific local coordinate framework to evaluate axial normal stresses along the insertion axes of individual screws [31]. This approach enables a more direct assessment of screw–bone interface loading associated with screw loosening and pull-out in elderly patients [13, 31, 32].

This study aims to provide a biomechanical rationale for a novel Hybrid LCP designed to overcome the limitations of conventional single-diameter systems, demonstrating how targeted stiffness modulation can optimize load redistribution and reduce screw pull-out risks in elderly clavicle fractures.

## Methods

This study was a retrospective, image-based biomechanical investigation using computed tomography (CT) data. All imaging data were anonymized prior to analysis and provided in accordance with institutional data protection policies. The research protocol was approved by the Institutional Review Board (IRB) of Chung-Ang University Gwangmyeong Hospital (Approval No. 2601-300-010).

### Finite element model construction

The three-dimensional clavicle model was generated from high-resolution CT images of a 73-year-old female patient. Image segmentation and reconstruction were performed using Mimics (version 19.0, Materialise NV, Leuven, Belgium), followed by geometric smoothing and repair in ANSYS SpaceClaim (ANSYS 2022 R2, Canonsburg, PA, USA).

**Fig. 1:**
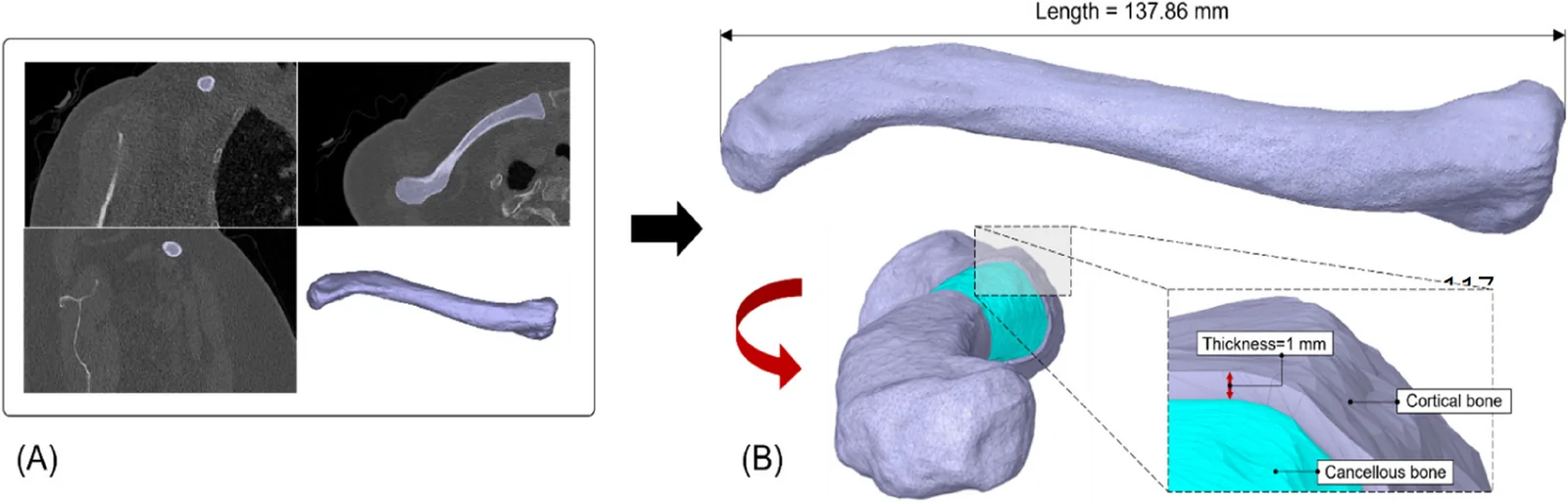
The 3D modeling of the elderly clavicle for finite element analysis. **A** 3D reconstruction of the clavicle morphology from CT images of a 73-year-old female patient using Mimics (Materialise, Leuven, Belgium). **B** Computational segmentation of cortical and cancellous bone using ANSYS SpaceClaim; the cortical shell thickness was specifically defined as 1.0 mm to represent elderly bone thinning

To accurately represent elderly bone morphology, the clavicle was modeled as a two-layer structure consisting of cortical and cancellous bone. The overall workflow for clavicle reconstruction and cortical–cancellous segmentation is illustrated in Fig. 1.

Andermahr et al. [12] reported a mean cortical thickness of 2.05 ± 0.29 mm at the clavicular midshaft in normal clavicles. In the present study, a uniform cortical thickness of 1.0 mm was assigned to represent a severely osteoporotic condition. This conservative modeling approach was adopted to rigorously evaluate implant performance under extreme age-related bone thinning. The internal region was filled with cancellous bone modeled as a solid volume, and continuity between cortical and cancellous regions was enforced by node sharing to ensure numerical continuity and anatomical fidelity [27]. A transverse midshaft fracture was simulated by introducing a 2.5-mm osteotomy gap at the center of the clavicle. This configuration was selected to evaluate the plate’s performance in its primary intended application: the stabilization of central diaphyseal fractures, thereby eliminating direct load transfer or frictional contact between fracture surfaces [25].

### Implant geometry and assembly

Four fixation constructs were evaluated: a conventional 3.5-mm locking compression plate, a curved 2.7-mm plate, a straight 2.7-mm plate, and the proposed Hybrid LCP. All plates and screws were constructed using manufacturer-provided CAD data to ensure geometric accuracy. The curved 2.7-mm plate geometry was based on a globally accepted commercial design, whereas the 3.5-mm plate, straight 2.7-mm plate, and Hybrid LCP were supplied by a domestic medical device manufacturer.

**Fig. 2:**
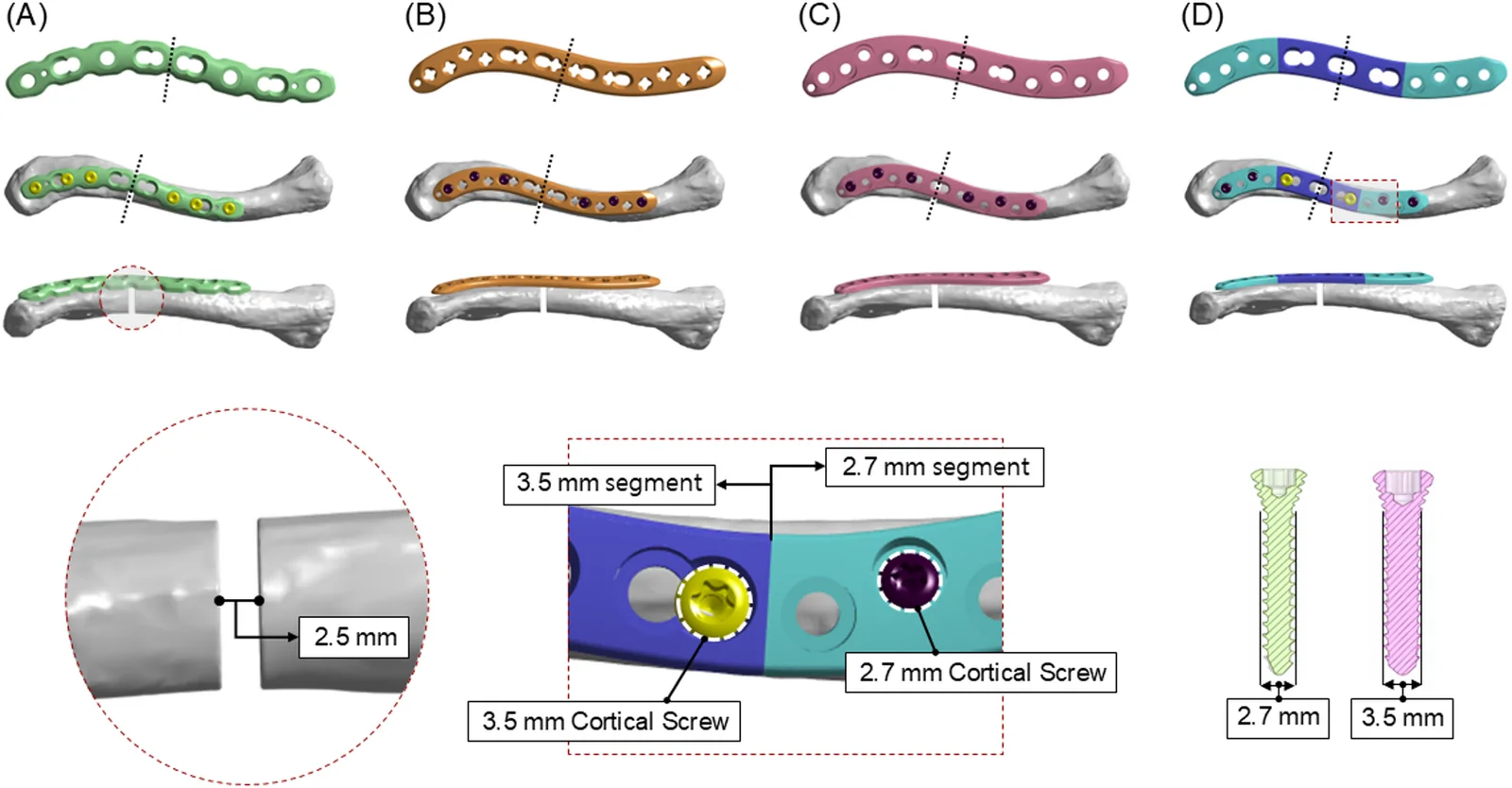
Geometric configurations of the four plate designs, fracture model generation, and fixation constructs used in the finite element analyses. **A** 3.5-mm plate. **B** Curved 2.7-mm plate. **C** Straight 2.7-mm plate. **D** Hybrid locking compression plate (Hybrid LCP). For all constructs, a 2.5-mm transverse fracture gap was created at the center of the plated region. The Hybrid LCP consisted of a central 3.5-mm segment and lateral 2.7-mm segments

All locking screws were modeled to precisely match the corresponding plate systems in terms of diameter and thread profile. The Hybrid LCP combined a 3.5-mm central plate segment with 2.7-mm lateral segments to create a spatially graded stiffness distribution along the construct. This configuration was selected based on the biomechanical principle that construct stiffness influences the mechanical environment of fracture healing in locking-plate fixation [33]. Accordingly, 3.5-mm screws were positioned in the central region adjacent to the fracture site, whereas 2.7-mm screws were placed in the lateral portions of the plate. Plate positioning was determined based on clinical consultation with an experienced orthopedic surgeon and aligned along the superior surface of the clavicle. The geometric configurations of the four plate systems and their clinically optimized placements on the clavicle model are shown in Fig. 2.

### Material properties

All materials were assumed to be linear elastic, homogeneous, and isotropic to ensure numerical stability and consistency with prior biomechanical studies. Material properties were assigned based on values reported in the literature [25, 26] and are summarized in Table 1. The plates and screws were modeled using the mechanical properties of Ti–6Al–4 V titanium alloy to reflect commonly used clinical implant materials.

**Table 1:** Material properties of the components used in the finite element analysis

Component	Young’s modulus (MPa)	Poisson’s ratio
Cortical bone	17,000	0.3
Cancellous bone	1,000	0.3
Ti-6Al-4 V	110,000	0.34

### Mesh generation and convergence analysis

All finite element models were discretized using 10-node quadratic tetrahedral elements (SOLID187). A global element size of 0.5 mm was applied throughout the model, while localized mesh refinement to 0.2 mm was implemented in regions with high stress gradients, including screw–bone interfaces, plate holes, and the fracture gap [34].

Mesh convergence analysis was conducted under the selected loading condition (cantilever bending). Element sizes were progressively reduced from 2.0 mm to 0.5 mm, and changes in maximum von Mises stress were monitored. Minimal variation was observed across successive refinements, indicating early numerical convergence of the model. The final mesh configuration was selected to balance computational efficiency and result reliability. Detailed mesh statistics for each model are provided in Table 2.

**Table 2:** Number of nodes and elements for the final assembled FE models

	Element number	Node number	Element type
Intact model	95,253	166,061	10-node quadratic tetrahedron (SOLID187)
3.5-mm Plate model	1,159,877	1,770,287	
Curved 2.7-mm Plate model	708,609	1,143,544	
Straight 2.7-mm Plate model	673,454	1,082,146	
Hybrid LCP model	833,599	1,307,946	

Intact model refers to the bone-only model without any implants; Plate models represent the final assembled FE models, including the clavicle, plate, and screws

### Contact definitions and boundary conditions

To simulate the mechanical behavior of a fully stabilized locking construct, bonded contact was assigned between the screw heads and plate holes, representing a fully constrained locking interface [27, 34]. Bonded contact was also applied at the screw–bone and plate–bone interfaces. These idealized contact definitions were applied consistently across all fixation models to isolate the comparative influence of plate geometry and stiffness distribution and to eliminate nonlinear variability associated with frictional sliding, contact separation, and interface micromotion [35, 36]. Accordingly, the resulting interface responses represent idealized comparative conditions rather than direct simulations of physiological implant–bone contact. The medial (sternal) end of the clavicle was fully constrained in all translational and rotational degrees of freedom to establish a standardized cantilever-type boundary condition, consistent with previously reported clavicle finite element models [25, 27, 34]. This idealized constraint was applied identically to all fixation constructs to enable direct comparison of their relative mechanical responses.

To accurately model the load transfer mechanism, the screw threads were explicitly retained rather than simplified. This geometric fidelity allows a more realistic representation of the screw–bone contact geometry and load-transfer pathway compared with simplified screw models. A bonded contact formulation was utilized to simulate a stable locking interface and ensure numerical convergence. Furthermore, local mesh refinement was specifically applied at the screw–bone interface and other high-gradient regions. Additional refinement below the selected mesh density produced negligible changes in the extracted peak stress/strain values, confirming mesh-independent results.

### Loading conditions

Standardized loading conditions were defined based on previously validated protocols [5, 25, 34]. All loads were applied to the lateral (acromial) end of the clavicle, and three independent loading scenarios were evaluated:

1. Axial compression: 100 N applied along the longitudinal axis of the clavicle, which was geometrically defined as the global straight-line vector connecting the centroid of the medial (sternal) end surface to the centroid of the lateral (acromial) end surface.
2. Cantilever bending: 100 N applied in the superoinferior direction.
3. Axial torsion: 1 N·m torsional moment applied about the clavicular long axis.

**Fig. 3:**
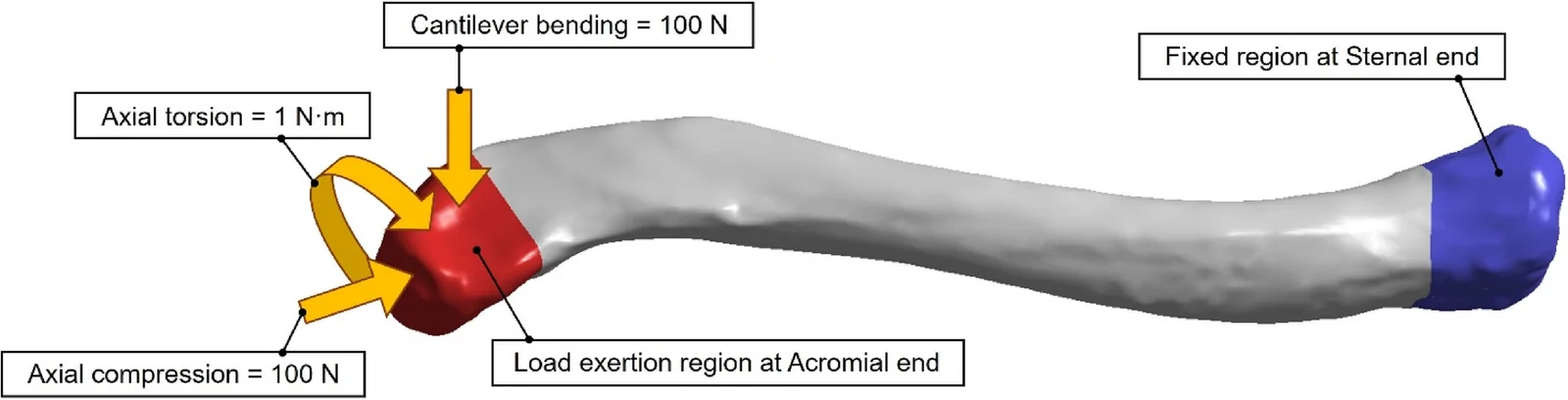
Boundary and loading conditions for finite element analysis. The sternal end of the clavicle is rigidly fixed. The acromial end is subjected to three standardized loads: axial compression (100 N), cantilever bending (100 N), and axial torsion. Note: The compression vector in Fig. 3 is aligned with the defined global longitudinal axis connecting the medial and lateral centroids; its apparent perpendicularity to the acromial surface is an artifact of the 3D perspective viewpoint

The applied boundary constraints and loading directions for all three standardized loading conditions are schematically illustrated in Fig. 3.

### Outcome measures and data processing

Three outcome measures were used to evaluate construct performance. First, von Mises stress distributions within the plates were analyzed to assess overall structural integrity and identify stress concentration regions. Second, screw–bone interface loading was quantified by calculating axial normal stress along the screw insertion axis. Third, peak stress and strain responses in the cortical and cancellous bone regions surrounding the fixation sites were extracted to characterize the modeled peri-implant bone-loading environment under each loading condition.

**Fig. 4:**
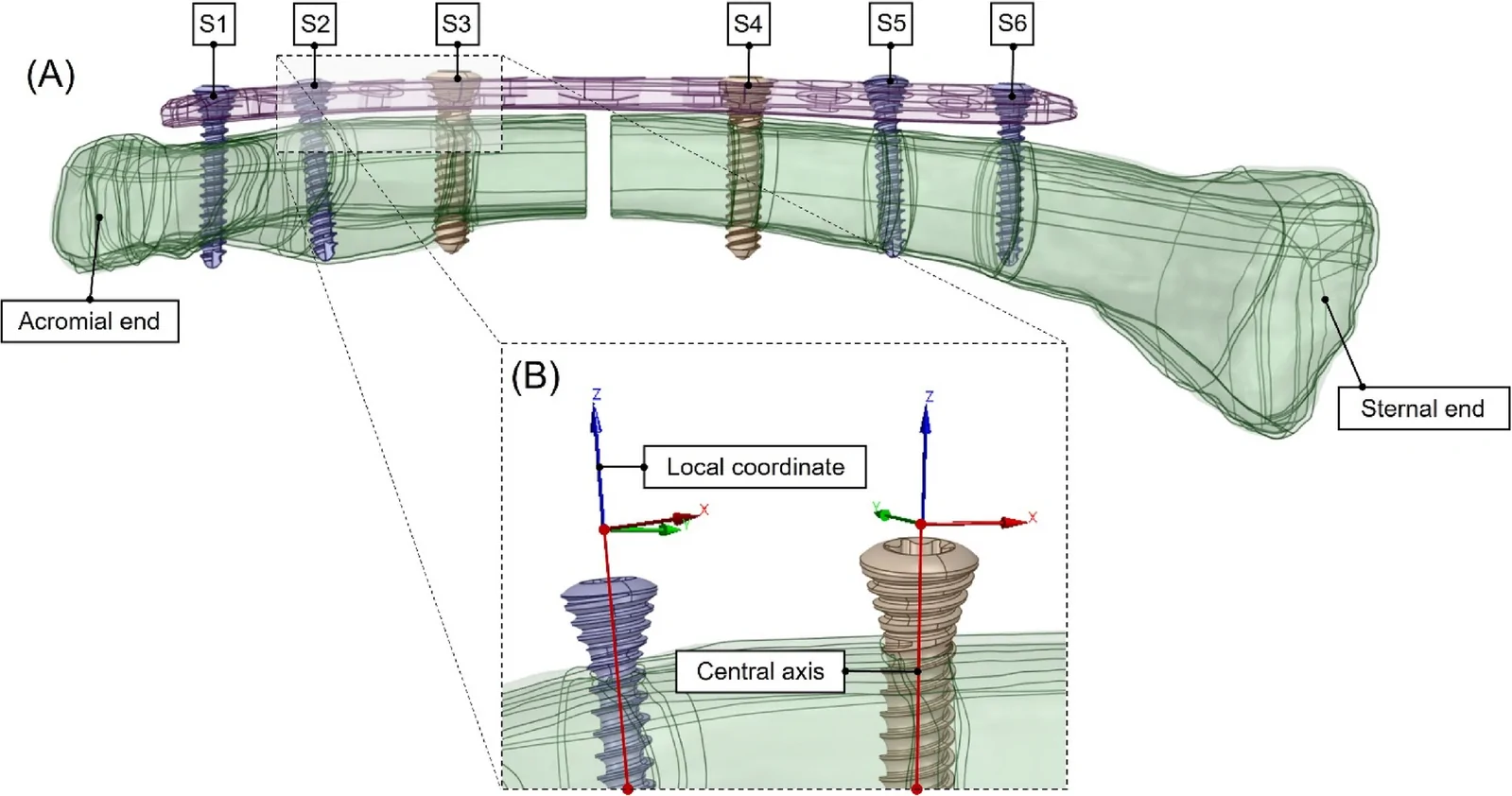
Definitions of the screw-specific local coordinate systems and numbering sequence. **A** A translucent assembly view illustrates the numbering of six screws (S1–S6) positioned from the acromial to the sternal end. **B** Magnified views demonstrate the alignment of the local coordinate axes with the screw insertion direction

To achieve this, a screw-specific local coordinate system was defined for each screw, with the local Z-axis aligned with the screw’s longitudinal axis. The positive Z-direction corresponded to the screw withdrawal (pull-out) direction. This local coordinate-based approach enabled isolation of clinically relevant tensile and compressive stress components directly associated with screw loosening and pull-out, independent of screw orientation [31, 32]. This screw-specific local coordinate system and the corresponding screw numbering scheme are illustrated in Fig. 4, demonstrating the alignment of the local Z-axis with the screw insertion direction for axial stress extraction. von Mises stresses were extracted directly from the screw-hole regions (S1–S6) to compare localized stress concentrations among the four fixation constructs.

Because the present study employed a single deterministic finite element model for each fixation construct without repeated independent samples, the computational outputs were treated as descriptive results, and inferential statistical testing was not applicable. For all relative comparisons between the Hybrid LCP and the curved 2.7-mm plate, percentage change was calculated as [(Hybrid LCP − curved 2.7-mm plate)/curved 2.7-mm plate] × 100. Under this convention, negative values indicate lower values in the Hybrid LCP, whereas positive values indicate higher values.

## Results

Finite element analyses were successfully completed for the intact clavicle model and all four fixation constructs under the three standardized loading conditions. All results were obtained using the converged mesh configurations described in the Methods section.

### von mises stress distribution in the plates

Distinct differences in von Mises stress distribution patterns were observed among the four plate models across all loading conditions. Among the selected loading scenarios, cantilever bending produced the highest stress levels.

Under cantilever bending, the curved 2.7-mm plate exhibited the highest maximum von Mises stress (637.7 MPa), followed by the 3.5-mm plate (629.5 MPa) and the straight 2.7-mm plate (548.1 MPa). In contrast, the Hybrid LCP demonstrated a substantial reduction in maximum von Mises stress (453.4 MPa) compared with both 2.7-mm plate systems and demonstrated an enhanced capacity for stress reduction, resulting in lower peak stress values than the 3.5-mm plate (Table 3).

**Table 3:** Summary of von Mises stresses for the four plate models (MPa)

	Cantilever bending			Axial compression			Axial torsion		
	Max.	Min.	Avg.	Max.	Min.	Avg.	Max.	Min.	Avg.
3.5-mm plate	629.5	140.0	195.9	77.6	17.2	26.2	40.4	9.0	13.6
Curved 2.7-mm plate	637.7	141.7	175.9	83.0	13.9	23.1	37.8	8.7	11.8
Straight 2.7-mm plate	548.1	121.8	154.1	74.3	16.5	20.3	28.5	6.3	7.3
Hybrid LCP	453.4	100.8	139.2	61.8	13.7	21.1	23.0	5.1	7.4

The Hybrid LCP consists of a 3.5-mm central portion and 2.7-mm lateral portions

A similar trend was observed under axial compression and axial torsion. Across all loading scenarios, the Hybrid LCP consistently reduced peak von Mises stress relative to the 2.7-mm plate systems while maintaining stable construct-level mechanical behavior. Mean von Mises stress values demonstrated a different pattern, with the 2.7-mm plates exhibiting lower average stresses than the 3.5-mm plate, reflecting differences in overall construct stiffness and von Mises stress distribution (Table 3).

**Fig. 5:**
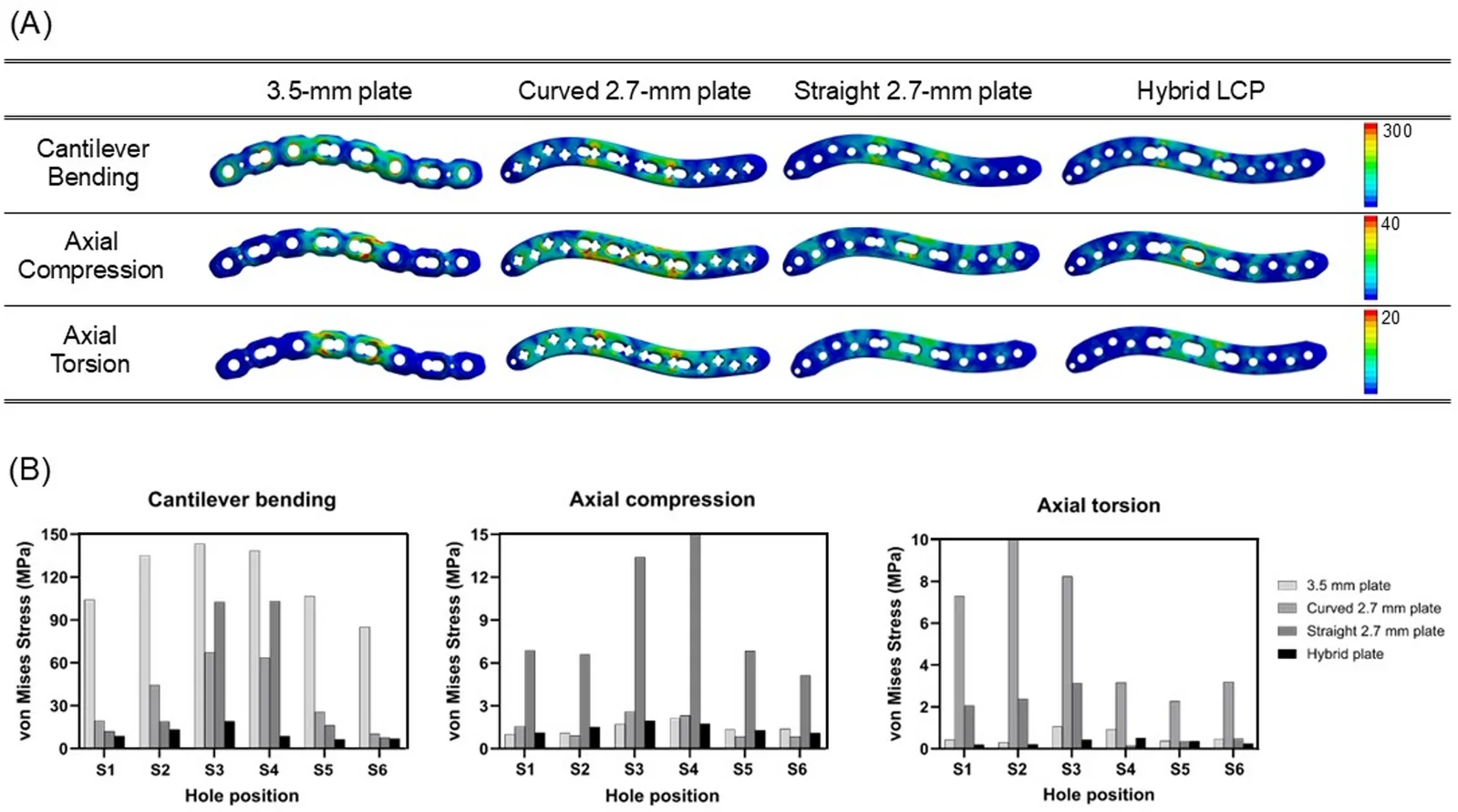
Plate von Mises stress distributions and screw-hole region stress responses under cantilever bending, axial compression, and axial torsion loading conditions. **A** Von Mises stress distributions of the four plate designs under the three loading conditions. The contour ranges were standardized within each loading condition as 0–300 MPa for cantilever bending, 0–40 MPa for axial compression, and 0–20 MPa for axial torsion to facilitate spatial comparison among the four plate models. Values exceeding the displayed upper limits appear as saturated regions, while the complete maximum von Mises stress values are reported quantitatively in Table 3. **B** von Mises stresses measured at the screw-hole regions (S1–S6) under cantilever bending, axial compression, and axial torsion

Across all models and loading conditions, von Mises stress concentrations were consistently localized around the screw holes adjacent to the fracture site, particularly at positions S3 and S4 (Fig. 5). For visualization purposes, the contour ranges were standardized within each loading condition to facilitate spatial comparison among the four plate models; values exceeding the displayed upper limits appear as saturated regions, while the complete maximum stress values are provided in Table 3. Minimal stress accumulation was observed at the terminal regions of the plates.

To quantitatively evaluate these stress patterns, von Mises stresses at the screw-hole regions were further analyzed for all four models (Fig. 5). As illustrated in Fig. 5, the Hybrid LCP demonstrated a markedly more uniform von Mises stress profile, effectively mitigating the peak von Mises stress concentration at the central screw-hole segments (S3 and S4) compared with the conventional 2.7-mm and 3.5-mm systems.

### Axial normal stress at the screw–bone interface

**Fig. 6:**
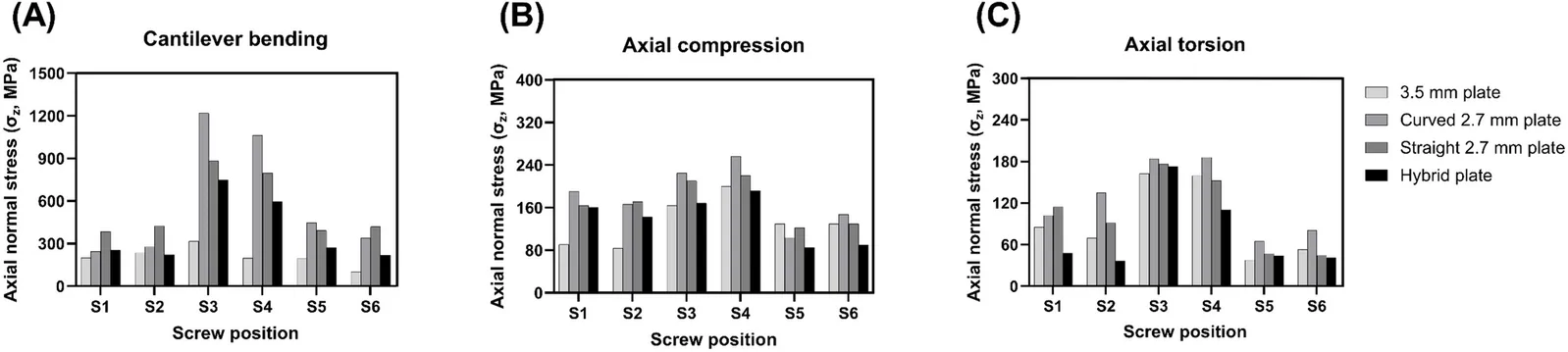
Quantitative comparison of screw axial normal stresses across the four plate models. Stresses were evaluated at each screw position (S1–S6) under (**A**) cantilever bending, (**B**) axial compression, and (**C**) axial torsion. Localized peak values exceeding the yield range of Ti–6Al–4 V are outside the physically reliable range of the linear-elastic material formulation and should be interpreted only as qualitative indicators of stress-prone regions, rather than as absolute material failure values

Axial normal stresses along the screw insertion axis were extracted using screw-specific local coordinate systems. Quantitative results for all screw positions (S1–S6) are summarized in Table 4, while complete distributions for all screws are presented in Fig. 6.

**Table 4:** Axial normal screw stresses (MPa) at all screw positions (S1–S6) under three standardized loading conditions

Loading condition	Screw position	3.5-mm plate	Curved 2.7-mm plate	Straight 2.7-mm plate	Hybrid LCP	% Change (Hybrid relative to curved 2.7-mm plate)
Cantilever bending	S1 (Acromial end)	200.3	244.6	385.2	254.1	+ 3.9%
	S2	234.4	279.8	422.5	220.9	-21.1%
	S3 (Fracture-side)	316.4	1218.1	881.7	794.2	-34.8%
	S4 (Fracture-side)	197.9	1063.3	797.1	593.9	-44.1%
	S5	194.0	447.8	392.0	270.5	-39.6%
	S6 (Sternal end)	103.5	339.7	418.9	219.9	-35.3%
Axial compression	S1 (Acromial end)	90.6	190.9	163.7	160.3	-16.0%
	S2	83.6	166.5	170.9	142.4	-14.5%
	S3 (Fracture-side)	164.5	224.9	210.4	168.4	-25.1%
	S4 (Fracture-side)	199.7	256.7	220.6	192.0	-25.2%
	S5	129.7	103.3	122.0	85.4	-17.3%
	S6 (Sternal end)	129.7	147.4	129.8	90.2	-38.8%
Axial torsion	S1 (Acromial end)	85.0	102.1	114.3	47.9	-53.1%
	S2	69.6	135.0	91.3	36.3	-73.1%
	S3 (Fracture-side)	162.6	184.0	176.1	172.5	-6.3%
	S4 (Fracture-side)	159.4	186.2	152.5	109.8	-41.0%
	S5	37.6	64.6	46.8	44.1	-31.7%
	S6 (Sternal end)	53.1	80.8	44.4	41.3	-48.9%

S1 and S6 represent the lateral and medial ends, respectively. S3 and S4 are adjacent to the fracture site. Percentage change was calculated as [(Hybrid LCP − curved 2.7-mm plate)/curved 2.7-mm plate] × 100. Negative percentage values indicate lower stress in the Hybrid LCP, whereas positive percentage values indicate higher stress. This percentage-change convention is independent of the axial-stress sign convention, in which positive stress denotes the withdrawal (pull-out) direction and negative stress denotes compression

#### Cantilever bending

Under cantilever bending, pronounced stress concentrations were observed at screws adjacent to the fracture site. The curved 2.7-mm plate demonstrated extreme axial normal stresses at S3 (1218.1 MPa) and S4 (1063.3 MPa). Elevated stresses were also observed in the straight 2.7-mm plate at the same locations (S3: 881.7 MPa; S4: 797.1 MPa).

In comparison, the Hybrid LCP reduced axial normal stresses at the fracture-adjacent screws, with values of 794.2 MPa at S3 and 593.9 MPa at S4. The 3.5-mm plate exhibited the lowest axial normal stresses at these positions (S3: 316.4 MPa; S4: 197.9 MPa) (Fig. 6A). The peak axial normal screw stress reached 1218.1 MPa. Because this value exceeds the yield range of Ti–6Al–4 V, its absolute magnitude lies beyond the physically reliable range of the linear-elastic formulation, which does not account for yielding or post-yield stress redistribution. The value should therefore be interpreted as a comparative indicator of a stress-prone region rather than as a physically realizable elastic stress or a direct predictor of implant failure.

#### Axial compression

Under axial compression, all constructs demonstrated reduced stress magnitudes compared with cantilever bending. Nevertheless, stress concentration patterns remained consistent, with higher stresses localized near the fracture site.

At S3 and S4, the Hybrid LCP exhibited axial normal stresses of 168.4 MPa and 192.0 MPa, respectively. These values were lower than those observed in the curved 2.7-mm plate (S3: 224.9 MPa; S4: 256.7 MPa) and the straight 2.7-mm plate (S3: 210.4 MPa; S4: 220.6 MPa). At the plate ends (S1 and S6), the Hybrid LCP maintained relatively uniform stress levels, indicating consistent load transfer along the construct (Fig. 6B).

#### Axial torsion

Under axial torsion, differences in load distribution among the constructs became more pronounced. The Hybrid LCP demonstrated the lowest axial normal stresses at the lateral screw positions, with values of 47.9 MPa at S1 and 36.3 MPa at S2. These stresses were lower than those observed in the 3.5-mm plate (S1: 85.0 MPa; S2: 69.6 MPa) and the curved 2.7-mm plate (S1: 102.1 MPa; S2: 135.0 MPa). At the medial end (S6), the Hybrid LCP also exhibited the lowest axial normal stress (41.3 MPa) among all models, indicating efficient torsional load redistribution along the construct (Fig. 6C).

### Peak cortical and cancellous bone stress-strain responses

The analysis of the bone tissue surrounding the screws revealed that the Hybrid LCP effectively distributed the load, resulting in a marked reduction in bone strain. The Hybrid LCP exhibited lower peak cancellous bone strain than the curved 2.7-mm plate under all three standardized loading conditions. Peak cancellous bone strain was 6,300 µε for the Hybrid LCP and 12,700 µε for the curved 2.7-mm plate under cantilever bending, corresponding to a percentage change of − 50.4% in the Hybrid LCP (i.e., 50.4% lower). Comparable percentage changes were observed under axial compression (− 45.6%; 3,100 µε vs. 5,700 µε) and axial torsion (− 48.8%; 2,100 µε vs. 4,100 µε). These values represent descriptive computational comparisons under the evaluated loading conditions rather than inferential estimates or universally applicable clinical effect sizes. These reductions suggest a more favorable modeled peri-implant mechanical environment and may indicate a lower potential for localized cancellous bone overloading under the standardized computational conditions. A comprehensive comparison of the biomechanical responses under various loading conditions is summarized in Table 5.

**Table 5:** Peak cortical and cancellous bone stress-strain responses under different loading conditions

Loading condition	Plate model	Cortical bone (MPa / µϵ)	Cancellous bone (MPa / µϵ)
Cantilever bending	3.5-mm plate	50.8 / 3,900	12.0 / 11,900
	Curved 2.7-mm plate	51.0 / 4,700	11.5 / 12,700
	Straight 2.7-mm plate	50.6 / 4,100	9.8 / 10,800
	Hybrid LCP	50.2 / 3,000	5.8 / 6,300
Axial compression	3.5-mm plate	26.3 / 1,500	5.6 / 6,200
	Curved 2.7-mm plate	26.8 / 1,600	5.2 / 5,700
	Straight 2.7-mm plate	26.1 / 1,600	4.8 / 5,300
	Hybrid LCP	25.4 / 1,500	2.9 / 3,100
Axial torsion	3.5-mm plate	21.1 / 1,200	4.1 / 4,500
	Curved 2.7-mm plate	21.5 / 1,300	3.8 / 4,100
	Straight 2.7-mm plate	20.8 / 1,200	3.5 / 3,800
	Hybrid LCP	18.2 / 1,100	2.0 / 2,100

Stress values are in MPa; strain values are in microstrain (µϵ)

### Summary of mechanical performance

Overall, the Hybrid LCP reduced the extreme fracture-adjacent screw stress concentrations observed in the 2.7-mm plate systems under cantilever bending. Its screw stress response approached that of the 3.5-mm construct near the fracture site, while lower stresses were observed at several terminal screw positions under compression and torsion. The Hybrid LCP also exhibited lower peak plate von Mises stress than all conventional constructs across the evaluated loading conditions.

## Discussion

The present study biomechanically evaluated a stiffness-distribution–controlled Hybrid LCP designed to address the inherent trade-off between mechanical integrity and anatomical compatibility in osteoporotic midshaft clavicle fracture fixation. Using a finite element model incorporating severe age-related cortical thinning, we demonstrated that the Hybrid LCP mitigated the high plate and screw stress concentrations observed in conventional 2.7-mm plate systems, particularly near the fracture site. The Hybrid LCP exhibited lower peak plate von Mises stresses than the conventional 3.5-mm plate across the three evaluated loading conditions. Although its fracture-adjacent screw axial normal stresses remained higher than those of the 3.5-mm plate, they were consistently lower than those of the conventional 2.7-mm systems.

The observed reduction in plate stress and localized screw stress concentrations in the Hybrid LCP can be attributed to its nonuniform stiffness distribution. By incorporating a thicker 3.5-mm central segment near the fracture site, the Hybrid LCP increased the local section modulus and bending resistance at the mechanically most critical region. In contrast, the transition to thinner 2.7-mm segments toward the lateral and medial ends allowed more compliant load transfer along the clavicle, thereby reducing abrupt stiffness mismatches between the implant and osteoporotic bone. This graded stiffness configuration facilitated redistribution of bending and torsional loads away from fracture-adjacent screw holes, where peak stresses were consistently observed in uniform 2.7-mm plate systems. Excessive implant stiffness is clinically disadvantageous in osteoporotic bone, as it can induce stress shielding and non-physiological load transfer [37, 38]. The quantitative analysis of local von Mises stresses around the screw-hole regions (Fig. 5B) substantiates this von Mises stress redistribution pattern. Across all loading scenarios, the Hybrid LCP consistently maintained a more uniform von Mises stress profile compared with conventional plates, effectively mitigating localized stress peaks at the critical screw-hole segments (S3 and S4). This comparative data supports that the Hybrid LCP promoted a more uniform von Mises stress distribution regardless of the standardized loading mode, reinforcing its potential for enhanced structural integrity.

Although the 3.5-mm system provides robust fixation, its high rigidity and bulk may predispose elderly patients to peri-implant fractures and soft tissue irritation [16, 18, 19, 39]. Conversely, the 2.7-mm plate models demonstrated pronounced mechanical vulnerability under bending loads, with extreme localized axial screw stress concentrations under the linear elastic formulation. Beyond the reduction in hardware stress, the biomechanical advantage of the Hybrid LCP was also reflected in the reduced cancellous bone strain observed in the surrounding bone. As summarized in Table 5, the 50.4% reduction in peak cancellous bone strain under cantilever bending was highlighted because this loading condition produced the highest overall stress levels. Comparable reductions were also observed under axial compression and axial torsion (45.6% and 48.8%, respectively) compared with the conventional curved 2.7-mm plate. These reductions suggest a more favorable modeled peri-implant mechanical environment and may indicate a lower potential for localized cancellous bone overloading under the standardized computational conditions. These findings indicate that low-profile 2.7-mm plates may lack sufficient mechanical capacity for unstable fracture patterns in osteoporotic bone [21].

In contrast to uniform single-diameter constructs, the Hybrid LCP achieved a favorable balance between mechanical integrity and anatomical compatibility [40]. The Hybrid LCP effectively reduced axial normal stress concentrations at fracture-adjacent screws compared with conventional 2.7-mm plate systems, while maintaining stress levels approaching those of the 3.5-mm system. Notably, under axial torsion, the Hybrid LCP exhibited efficient load redistribution with lower axial screw stresses at both lateral and medial plate ends, suggesting potential advantages in resisting rotational loads encountered during early postoperative shoulder motion [33]. These findings support the concept that strategic reinforcement of mechanically critical regions, rather than uniform plate thickening, was associated with more favorable mechanical responses in the osteoporotic bone model. This aligns with recent finite element studies demonstrating that screw positioning plays a critical role in determining von Mises stress distribution and preventing fixation failure [13, 32, 41].

From a clinical perspective, these findings underscore the limitations of conventional single-diameter plate systems in elderly clavicle fracture fixation. Neither uniformly rigid nor uniformly low-profile constructs adequately address the combined challenges of compromised bone quality and soft tissue tolerance in geriatric patients. Indeed, recent clinical evidence indicates that hardware removal rates remain substantial due to persistent soft tissue irritation, even with the introduction of newer anatomical plate designs [42]. Consequently, the present results suggest that stiffness-distribution–controlled plate designs may offer a potentially useful design concept by balancing localized mechanical demands with improved anatomical compatibility [43]. Specifically, the incorporation of low-profile 2.7-mm segments reduces the implant’s physical volume in the lateral clavicle, where soft tissue coverage is often compromised. While soft tissue interaction was not directly simulated in this study, minimizing implant prominence is widely recognized as a geometric advantage that may potentially mitigate skin irritation and hardware-related discomfort compared with the bulkier conventional 3.5-mm systems. Although clinical validation is required, this design concept may represent a promising direction for age-specific fracture fixation [44]. Because the present Hybrid LCP was constructed by combining manufacturer-provided 3.5-mm and 2.7-mm plate geometries, this study focused on demonstrating the biomechanical feasibility of the stiffness-distribution concept rather than quantitatively optimizing sectional stiffness parameters. Future studies incorporating quantitative sectional stiffness analyses and geometric optimization may further refine this design strategy.

Physical testing of osteoporotic clavicles is often complicated by substantial inter-specimen variability in bone quality and morphology. The controlled computational framework used in the present study enabled the influence of plate stiffness distribution to be evaluated under standardized severe cortical-thinning conditions while minimizing biological variability. A healthy-bone control model was not included because the study was specifically designed to compare the mechanical performance of the four fixation constructs under compromised bone conditions, in which reduced implant anchorage and localized peri-screw overloading are particularly relevant concerns [13, 32].

Several limitations of this study should be acknowledged. First, the cortical and cancellous bone components were modeled as linear elastic, homogeneous, and isotropic materials, with the cancellous bone represented as a continuum solid. This approximation does not reproduce the heterogeneous trabecular microarchitecture or nonlinear elastoplastic behavior of osteoporotic bone and may therefore limit the physiological accuracy of the localized screw–bone stress and strain values. In addition, the linear-elastic formulation does not account for localized yielding, trabecular crushing, or post-yield stress redistribution in bone. The implant components were likewise modeled using linear-elastic material properties; therefore, localized stresses exceeding the material yield range should be interpreted as comparative indicators rather than as physically realizable post-yield stresses. Nevertheless, because identical material assumptions were applied to all constructs, the relative comparisons among the four plate configurations remain informative. Future studies should incorporate heterogeneous and elastoplastic bone models, drawing on established elastoplastic modeling approaches for porous mineralized tissues [45], to improve the representation of localized bone yielding and crushing. Nonlinear implant material formulations combined with experimental or sensor-informed validation should also be considered to evaluate post-yield plate deformation [46].

Second, the plate–bone and screw–bone interfaces were modeled using fully bonded contact conditions. Although these simplifications were intentionally adopted to isolate the mechanical effects of plate stiffness distribution, they represent idealized approximations of in vivo interface behavior. A fully bonded interface prevents relative motion and may therefore underestimate interface micromotion and alter local load-transfer pathways. A previous patient-specific finite element analysis demonstrated that assumptions regarding bone–bone and bone–plate friction can affect stress transfer within a reconstructed bone–implant system [47]. Plate–bone contact may also alter the effective working length, interfragmentary motion, and maximum plate stress of a fixation construct [35]. Therefore, the quantitative screw axial normal stress profiles reported in this study should be interpreted as idealized comparative baselines, and the potential implications of unmodeled interface micromotion, screw loosening, and fatigue-related fixation failure should be considered when extrapolating these findings [13, 32]. Future analyses incorporating frictional contact, contact separation, and interface micromotion are required to more realistically evaluate screw loosening and localized bone failure.

Third, the medial (sternal) end of the clavicle was fully constrained in all translational and rotational degrees of freedom to establish a standardized cantilever-type boundary condition. Although this simplified condition enabled direct comparison among the four fixation constructs under identical loading conditions, it does not reproduce the physiological translational and rotational compliance of the sternoclavicular joint, ligamentous restraint, or articular contact. Consequently, the rigid constraint may increase the apparent stiffness of the bone–implant construct and affect, or potentially overestimate, the calculated absolute stress magnitudes. Because the same boundary condition was applied consistently to all four constructs, the relative mechanical comparisons among the plate configurations remain informative within the present standardized computational framework. However, the absolute stress values should be interpreted cautiously and should not be regarded as direct predictions of in vivo stresses. Future models incorporating ligamentous restraint, articular contact, and physiological translational and rotational compliance at the sternoclavicular joint are warranted to improve the physiological fidelity of the model.

Fourth, a uniform cortical thickness of 1.0 mm, corresponding to approximately 49% of the reported mean cortical thickness at the clavicular midpoint (2.05 ± 0.29 mm), was selected to represent a standardized severe cortical-thinning condition [12]. Although this assumption enabled a controlled comparison among the fixation constructs, it does not capture regional variations in clavicular cortical morphology, cortical thickness, or bone quality.

Fifth, the analysis focused on static loading scenarios to assess initial fixation integrity, and cyclic loading or fatigue-related failure mechanisms were not explicitly evaluated. Reduced implant anchorage is a recognized concern in osteoporotic fixation [13], and peri-screw strain has been shown to predict fatigue failure in osteoporotic plated constructs [32]. In addition, the fracture gap was positioned exclusively at the center of the reinforced 3.5-mm segment to evaluate the intended application of the Hybrid LCP to midshaft fractures, which account for the majority of clavicle fractures [1, 2]. Consequently, the present findings should not be generalized to fractures located outside the reinforced central segment.

Finally, the finite element model was reconstructed from a single CT dataset of a 73-year-old female. Given the substantial inter-individual variability in bone quality, regional morphology, and cortical thickness among elderly individuals, a single-patient model cannot comprehensively represent the broader geriatric population. Furthermore, no formal sensitivity analysis was performed to evaluate the effects of variations in cortical thickness, bone elastic properties, load magnitude and direction, screw-thread mesh density, or implant–bone contact definitions. Therefore, although comparable reductions in peak cancellous bone strain were observed across the three standardized loading conditions, the exact numerical percentages may vary under different anatomical, material, loading, mesh, or contact assumptions. The reported values should thus be interpreted as relative computational trends rather than as absolute in vivo metrics or direct predictors of clinical failure. Future studies should incorporate systematic parameter variation, statistical shape modeling, multiple patient-derived models, cyclic fatigue testing [32], and experimental or sensor-informed preclinical validation [46] to assess the robustness and generalizability of the observed comparative trends. Mechanical testing using synthetic bone models may provide an additional framework for validating the absolute fidelity of the computational predictions.

## Conclusion

Under the standardized computational conditions evaluated in this study, the stiffness-distribution–controlled Hybrid LCP exhibited lower fracture-adjacent screw axial stresses than the conventional 2.7-mm plate systems and lower peak plate von Mises stresses than the conventional 3.5-mm plate. The Hybrid LCP showed 50.4%, 45.6%, and 48.8% lower peak cancellous bone strain than the curved 2.7-mm plate under cantilever bending, axial compression, and axial torsion, respectively. These findings indicate that central reinforcement combined with lower-profile lateral segments may provide a more balanced modeled mechanical environment for osteoporotic midshaft clavicle fixation. However, the findings were derived from a single deterministic finite element model and should be interpreted as comparative mechanical trends rather than absolute in vivo values or estimates of clinical risk reduction. Further sensitivity analyses, multi-patient modeling, experimental validation, and clinical investigation are required before the generalizability and clinical applicability of the proposed design can be established.

## Data Availability

The datasets used and analyzed during the current study available from the corresponding author on reasonable request.

## Abbreviations

Hybrid LCP: Stiffness-distribution-controlled hybrid locking compression plate
FEA/FE: Finite Element Analysis / Finite Element
CT: Computed Tomography
CAD: Computer-Aided Design
Ti–6Al–4V: Titanium alloy (Titanium-Aluminum-Vanadium)
MPa: Megapascal
µε: Microstrain
